# The independent effect of living in malaria hotspots on future malaria infection: an observational study from Misungwi, Tanzania

**DOI:** 10.1186/1475-2875-13-445

**Published:** 2014-11-21

**Authors:** Jacklin F Mosha, Hugh JW Sturrock, Joelle M Brown, Ramadhani Hashim, Gibson Kibiki, Daniel Chandramohan, Roland D Gosling

**Affiliations:** National Institute for Medical Research (NIMR), Mwanza Medical Research Centre, Mwanza, Tanzania; The Global Health Group, University of California, San Francisco, CA USA; Department of Epidemiology and Biostatistics, University of California San Francisco, San Francisco, CA USA; Mwanza Intervention Trials Unit, Mwanza, Tanzania; Kilimanjaro Clinical Research Institute and Kilimanjaro Christian Medical College, Kilimanjaro, Moshi Tanzania; Faculty of Infectious and Tropical Diseases, London School of Hygiene and Tropical Medicine, London, UK

**Keywords:** Malaria, Transmission, Hotspots, Risk factor, Serology, PCR, Africa, *Plasmodium falciparum*

## Abstract

**Background:**

As malaria transmission declines, continued improvements of prevention and control interventions will increasingly rely on accurate knowledge of risk factors and an ability to define high-risk areas and populations at risk for focal targeting of interventions. This paper explores the independent association between living in a hotspot and prospective risk of malaria infection.

**Methods:**

Malaria infection status defined by nPCR and AMA-1 status in year 1 were used to define geographic hotspots using two geospatial statistical methods (SaTScan and Kernel density smoothing). Other malaria risk factors for malaria infection were explored by fitting a multivariable model.

**Results:**

This study demonstrated that residing in infection hotspot of malaria transmission is an independent predictor of malaria infection in the future.

**Conclusion:**

It is likely that targeting such hotspots with better coverage and improved malaria control strategies will result in more cost-efficient uses of resources to move towards malaria elimination.

## Background

Transmission of malaria is highly heterogeneous even in areas of moderate transmission with clusters of households that are at consistently high levels of risk of malaria. These clusters, termed hotspots, are responsible for spread of malaria infection in the wet season [1]. As malaria transmission declines, prevention and control interventions will increasingly rely on accurate knowledge of risk factors and an ability to define high-risk areas and populations at risk for focal targeting of interventions. This could be useful in the allocation of limited resources to ensure areas that require them the most are given priority.

Several studies have documented individual and household risk factors associated with malaria infection. Some of the risk factors that have already been reported to be associated with malaria infection include type of housing [2–4], socio-economic status (SES) [5, 6], proximity to mosquito breeding sites [5–8], age, and sex [2, 5, 6, 9]. If the theory of malaria hotspots is true, that infection clusters in small spatial scales, then residing in a hotspot should be an important independent risk factor for individual level risk of malaria infection as local mosquitoes are more likely to be infectious. Identification of malaria transmission hotspots are important in order to focus the control and elimination activities to appropriate geographic areas and also to select the appropriate population level interventions, such as indoor residual spray in addition to interventions targeted towards high risk groups. This paper examines the independent association between living in a malaria hotspot and future risk of malaria infection.

## Methods

### Study site

Misungwi district (lat 2.85000 S, long 33.08333 E) is located 60 km from Mwanza town in the northwest of Tanzania at an altitude of 1,178 m above sea level. Details of the study site have been previously described [10]. In brief, the district is rural with moderately intense malaria transmission; the overall prevalence of infection in the region is estimated to be 31.4% by microscopy in children six to 59 months of age [11].

### Study design

This is a cross sectional study, which was conducted twice in year 1 in 2010 and year 2 in 2011. The exposure of interest was whether a person resided in a malaria hotspot or not in the first year of the study. Hotspots were defined by SaTScan and Kernal density method using infection status derived using nested polymerase chain reaction (nPCR) and AMA-1 sero status in year 1, as previously reported [10]. The methods are explained briefly below. The outcome of interest for this analysis was infection status at the individual level by nPCR (infected/not infected) in the survey taken in the second year. In brief, the SaTScan software (SaTScan, version 8.2.1) used a spatial scan statistic using the Bernoulli model to identify clusters of significant high (hotspot) and low (coldspot) risk of infection [12]. Using the SaTScan method, SaTScan cold spots were coded as 0, hotspots as 1 and everything else as 0.5. The kernel method of household clustering of both nPCR and AMA-1-positive individuals was estimated using Kernel density smoothing. Kernel density estimates, for any given point, the density of events within a predefined window, with the influence of events weighted according to the distance from the centre of the window. The weight assigned to each event is derived from the kernel function applied. Details of these methods have been described previously [10]. Using the Kernel method, each household was assigned a value between 0 (least exposed households) and 1 (most exposed households). Households for which data were only available in the second year were assigned a hotspot score based on infection in neighboring households only.

### Data collection

A census of four villages in a single ward of the Misungwi district of Tanzania was carried out in the dry season, in two consecutive years 2010 and 2011 between the months of August and November. Trained interviewers administered a structured questionnaire to consenting household heads. Information gathered included morbidity, demographics and data on potential risk factors. Latitude and longitude of each household was taken using a GPS device. Data were recorded electronically using personalized digital assistants and were downloaded each evening of the survey onto a desktop computer to a master Microsoft access database. Also, every consenting individual was asked whether they slept under a long-lasting insecticidal-treated net (LLIN) the previous night. A finger-prick blood sample was collected and was spotted onto Whatman® standard 3 mm filter paper for parasite detection by nPCR and for serology analysis (AMA-1).

### Statistical analysis

Statistical analysis was performed using STATA (version 12, College Station, TX, USA). Mixed effect logistic regression models were used to evaluate whether residing in a hotspot in the first year was predictive of subsequent malaria infection in the second year, controlling for other potential risk factors for malaria infection. The model adjusted for possible clustering of malaria cases within households.

Summary contingency tables, graphs and scatter plots with lowess curves were used to explore the relationship with potential risk factors and the outcome (malaria infection defined by nPCR in the second year). How well the linear and quadratic terms of these variables fitted was also explored. Variables with a non-linear relationship to the outcome (age, SES, number of cattle sleeping outside the household, and number of people sleeping in the household) were categorized. Age was categorized into five groups: under four years, five to 15 years, 16–25 years, 26–35 years and over 36 years (Table 1); number of cattle into three groups: none, one to ten and ten + cattle. SES was based on wealth index, which was a weighted sum of data on household possessions and utilities, according to principal component analysis. SES was categorized by dividing wealth index into four wealth quartiles, from the poorest to the least poor. Principle component analysis was conducted using a set of household construction materials (wall material, roof material, floor material, presence of eaves, and whether windows were screened or not) to define household quality. The household quality index was categorized by dividing the index into tertiles. Presence of ponds, rice plantations, water in clay pots, old tires, garbage, and any kind of stagnant water around the house were considered a mosquito breeding site. The presence of breeding site within 100 m (which was manually checked by the study team) around the household was chosen as this is considered to be the distance with abundant vector densities, and also vector densities decline rapidly away from the breeding sites [9]. Euclidean (straightline) distance to health facility from each surveyed household was calculated using coordinates of the households and that of health facility the household attended. Guided by lowess curves distance to the health facility was categorized into four groups, <1 km, 1-2.5 km, 2.6–3.5 km, 3.6+ km.

**Table 1:** **Univariate analysis of potential risk factors for malaria infection in year 2, as measured by nPCR**

Variable	N = 3,246	N (%) with malaria n = 1,683	Crude OR* (95% CI)	Wald test P-value
**Age group inyears**				
0-4	824	349 [42.3]	1	
5-15	1,003	695 [69.3]	5.04 [3.82-6.63]	<0.001
16-25	445	235 [52.8]	2.00 [1.41-2.72]	<0.001
26-35	334	337 [44.8]	1.08 [0.76-1.56]	0.661
36+	547	253 [39.7]	0.87 [0.63-1.19]	0.382
**Sex**				
Female	1,896	912 [48.1]	1	
Male	1,350	771 [57.1]	1.38 [1.13-1.68]	0.002
**Sleep under ITN**				
No	291	191 [65.6]	1	
Yes	2,955	1,492 [50.5]	0.42 [0.27-0.66]	<0.001
**Wealth quartile**				
Poorest	610	337 [61.1]	(Per additional increase in wealth quartile) 0.69 [0.58-0.82]	<0.001
Very poor	887	492 [55.5]		
Less poor	930	478 [51.4]		
Least poor	819	340 [41.5]		
**Maternal education**				
None	1,545	930 [60.2]	1	
Primary/+	1,701	753 [44.3]	0.40 [0.28-0.57]	<0.001
**Breeding site**				
No	1,650	737 [44.7]	1	
Yes	1,596	959 [59.3]	2.53 [1.75-3.65]	<0.001
**Household quality**				
High	966	439 [45.4]	1	
Moderate	560	198 [35.4]	0.44 [0.26-0.75]	0.002
Poor	1,704	1,038 [60.9]	2.32 [1.54-3.50]	<0.001
**Indoor residual spraying**				
No	523	281 [53.7]	1	
Yes	2,723	1,402 [51.5]	0.98 [0.60-1.60]	0.925
**Number of cattle**				
0-0	1,368	676 [49.4]	1	
1-10	718	433 [60.3]	1.96 [1.20-3.21]	0.007
11+	1,160	574 [49.5]	0.68 [0.45-1.04]	0.075
**Distance to health facility**				
<1 km	712	242 [34.0]	(per additional increase in distance group to health facility) 2.01 [1.69-2.40]	<0.001
1-2.5 km	934	455 [48.7]		
2.6-3.5 km	971	556 [57.3]		
3.6+ km	611	424 [69.4]		
**Residence in a hotspot (SaTScan-nPCR)**				
Coldspot	792	319 [40.3]	1	
Other	1,728	864 [50.0]	1.40 [0.91-2.15]	0.125
Hotspot	726	500 [68.9]	4.44 [2.64-7.46]	<0.001
**Residence in a hotspot (SaTScan-AMA-1)**				
Coldspot	904	310 [34.3]	1	
Other	1,092	554 [50.7]	2.66 [1.71-4.13]	<0.001
Hotspot	1,250	819 [65.5]	5.87 [3.79-9.05]	<0.001
**Residence in a hotspot (Kernel-nPCR)**				
<14.9	804	390 [48.5]	1	
15-21.3	819	387 [47.2]	0.99 [0.60-1.64]	0.966
21.4-27.1	818	331 [40.5]	0.53 [0.32-0.88]	0.013
>27.1	805	575 [71.4]	3.45 [2.06-5.75]	<0.001
**Residence in a hotspot (Kernel-AMA-1)**				
<27.9	814	309 [38.0]	1	
28-38.9	811	409 [50.4]	2.21 [1.34-3.66]	0.002
39-53.0	814	428 [52.6]	2.61 [1.58-4.31]	<0.001
>53.0	807	537 [66.5]	5.15 [3.09-8.60]	<0.001

*OR = Odds ratio; adjusted for possible household clustering.

All variables were analysed individually for an association with the outcome (malaria by nPCR infection in year 2) using logistic regression. A household-level random effect was included to account for correlation between individuals within the same household. All variables showing evidence for a possible association with malaria risk (p < 0.1) were included in the preliminary main effect multivariate logistic regression model. A forward stepwise approach was then followed to exclude any variable that showed a lack of effect on malaria risk (p > 0.05). Hotspots defined by SaTScan and kernal are fitted in different multivariate models.

## Results

A total of 3,246 individuals participated and provided a blood specimen in year 2. This represents 85.4% of individuals in the community who were eligible to participate. The median age of the study population was 13 years (IQR = 5–30 years; range <1-99 years) and 41.6% were male. The uptake of vector control measures was high in the study communities; 91% of the study participants reported to be sleeping under an LLIN the previous night, and 82% of households had received IRS within the six months before the survey.

### Univariate analysis

Table 1 presents the results of the univariate associations with individual infection status in year 2. These univariate estimates were adjusted for possible household level clustering. Residing in hotspots defined by malaria infection and AMA-1 sero status were associated with higher odds of malaria infection. Children between the age of five and 15 years and males had significantly higher odds of malaria infection. Higher wealth status, use of LLIN and mother’s education were associated with lower odds of malaria infection. Households with poor quality of construction materials, presence of a breeding site near the household and greater distance to the health facility were associated with higher odds of malaria infection.

### Multivariate analysis

Table 2 presents results from the multivariable analysis to determine the independent risk of malaria infection associated with residing in a malaria hotspot, adjusting for other risk factors for malaria infection and for household clustering. Only residing in malaria infection hotspots, using SaTScan method to define hotspots, was predictive of increased odds of malaria infection in year 2. For example, individuals residing in infection hotspot defined by SaTScan were three times more likely to have malaria infection after controlling for other factors (OR 3.11; 95% CI 1.57, 6.18).

**Table 2:** Multivariate models* estimating the independent risk of malaria infection associated with residing in a malaria hotspot

Variable	N = 3,246	Adjusted OR** (95% CI)	Wald test P-value
**Residence in a hotspot (SaTScan-nPCR)**			
Coldspot	792	1	
Other	1,728	1.64 [0.96-2.81]	0.072
Hotspot	726	3.11 [1.57, 6.18]	<0.001
**Residence in a hotspot (SaTScan-AMA-1)**			
Coldspot	904	1	
Other	1,092	1.66 [0.79-3.48]	0.767
Hotspot	1,250	1.78 [0.91-3.46]	0.091
**Residence in a hotspot (Kernel-nPCR)**			
<14.9	804	1	
15-21.3	819	0.66 [0.35-1.04]	0.070
21.4-27.1	818	0.88 [0.46 -1.66]	0.690
>27.1	805	1.52 [0.87-2.66]	0.145
**Residence in a hotspot (Kernel-AMA-1)**			
<27.9	814	1	
28-38.9	811	0.70 [0.37-1.31]	0.264
39-53.0	814	0.65 [0.32-1.32]	0.237
>53.0	807	0.99 [0.49-2.00]	0.987

*The model for SaTScan and kernel were run separately, and were adjusted for the following variables: age, sex, mother’s education, breeding site, household quality, sleeping under LLIN, and distance from health facility.

**OR = Odds ratio; adjusted for possible household clustering.

However, hotspots defined by both nPCR and AMA-1 using kernel method did not appear to be independent risk factors for future malaria infection after controlling for other factors in the multivariable analysis, OR 1.52; 95% CI 0.87-2.66 and OR 0.99; 95% CI 0.49-2.00, respectively.

Apart from residing in a malaria infection hotspot being an independent risk factor for malaria infection in the second year, other factors were also independently associated with increased risk of malaria infection in the second year in all multivariable hotspot models. These were age, gender, mother’s education, using LLIN, presence of breeding sites, longer distance to a health facility, and lower quality of houses (Table 3).

**Table 3:** **SaTScan model of risk factors associated with malaria infection in year 2 in the multivariable analysis**

	SaTScan	
Variable	Adjusted OR (95% CI)	Wald test P-value
**Residence in a hotspot (nPCR)**		
Coldspot	1	
Other	1.64 [0.96-2.81]	0.072
Hotspot	3.11 [1.57, 6.18]	<0.001
**Residence in a hotspot (AMA-1)**		
Coldspot	1	
Other	1.66 [0.79-3.48]	0.767
Hotspot	1.78 [0.91-3.46]	0.091
**Age group in years**		
0-4	1	
5-15	5.04 [3.82-6.64]	<0.001
16-25	1.86 [1.33-2.64]	<0.001
26-35	1.23 [0.85-1.78]	0.262
36+	0.84 [0.62-1.16]	0.293
**Sex**		
Female	1	
Male	1.24 [1.01-1.53]	0.043
**Sleep under LLTN**		
No	1	
Yes	0.40 [0.25-0.63]	<0.001
**Household quality**		
High	1	
Moderate	0.71 [0.38-1.39]	0.090
Poor	1.53 [1.01-2.32]	0.044
**Mother education**		
None	1	
Primary/+	0.66 [0.45-0.95]	0.024
**Breading site**		
No	1	
Yes	1.59 [1.11-2.29]	0.011
**Distance to health facility**		
Per additional increase in distance	1	
Group to health facility	1.34 [1.03-1.76]	<0.031

Individuals in the age group of five to 15 years had more than five times the odds of infection than those who were in younger age group (OR 5.04; 95% CI 3.82-6.64). There was borderline evidence that the risk of malaria infection was higher for males than females (OR 1.24; 95% CI 1.01-1.53).

Individuals living within 100 meters of a mosquito breeding site had increased odds of malaria infection compared to those not living near a mosquito breeding site (OR 1.59; 95% CI 1.11-2.29). Individuals living in poor-quality households had increased odds of malaria infection compared to those who were living in good-quality households (OR 1.53; 95% CI 1.01-2.32). Distance to the health facility was strongly associated with risk of having malaria infection. Individuals residing in households based further from health facilities had increased odds of malaria infection (OR 1.34; 95% CI 1.03-1.76).

There was strong evidence that sleeping under LLIN was associated with decreased odds of infection (OR 0.40; 95% CI 0.25-0.63). Individuals whose mother had primary education or more had decreased odds of malaria infection compared to those individuals whose mother had not gone to school (OR 0.66; 95% CI 0.45-0.95).

Wealth, IRS, number of cattle, and number people sleeping in the house were not significantly associated with the risk of malaria infection in multivariate analyses.

## Discussion

This study demonstrates that residing in an infection hotspot of malaria transmission is an independent predictor of getting malaria infection in the future, adjusting for known risk factors for malaria infection. This could be due to individuals’ proximity to other infections in the area, as mosquitoes tend to stay within the same area, puts others in the area at higher risk. Equally, there could be other spatially clustered risk factors which haven’t been accounted for, and there is therefore residual clustering of infections. The independent effect of residing in hotspots of malaria infection was only found when SaTScan was used to identify hotspots. SaTScan analysis identified a single big central cluster of malaria hotspots and kernel analysis identified the central cluster that was identified by the SaTScan method and also identified other smaller clusters of malaria hotspots. Possible explanation could be there are factors that are influencing risk in the central hotspot that are not adjusted for. Whereas, factors that are explaining risk of malaria infection in other hotspots identified by kernel are included in the model and they have been adjusted. The data support the hypothesis that residing in a malaria hotspot is an independent predictor of future malaria risk, controlling for other known risk factors for malaria.

The observed increased risk of malaria infection in older children (age group five to 15 years) compared to children under five years of age may possibly be due to the fact that older children are more exposed to infectious mosquitoes as this age group tend to be more active and hence spend more time outside the household in late evening and early night. In previous studies malaria risk was reported to be high in younger children [1, 13, 14]. However, the increased risk of malaria infection in older children in this study could also be as a result of overall increase in LLIN coverage in younger children in the study communities and also in other parts of Tanzania after universal distribution of LLINs [15]. The same pattern of increased risk in older children has also been observed by other studies conducted in Tanzania [16, 17]. Likewise, the study used PCR for parasite detection rather than microscopy or RDT, therefore the likelihood of picking up more low density infections was much higher. It has been observed that decreasing transmission results in age escalation of infection to older children [18]. This could be due to slower development of naturally acquired immunity.

Living far from health facility has been associated with increase in malaria risk [19]. This study observed the same trend with malaria risk increasing with increasing distance from health facility. Individuals living near health facilities could be making more frequent trips to the health facilities and this might have resulted in more opportunities for health messages reinforcing proactive efforts to protect their health and of other family members and encourage early treatment, which is expected to clear infections completely. Individuals living far from health facilities could delay seeking prompt malaria treatment or easily choose to seek other alternative traditional treatment, which is ineffective and results in ongoing malaria transmission.

Environmental factors such as proximity of household to water bodies, bushes and stagnant water acting as breeding site for mosquitoes have been shown to be a major risk factor for malaria infection and transmission [2, 20–23]. Previous entomological studies have suggested that mosquitoes tend to have blood meals from humans that are in close proximity [9, 24]. Despite high coverage of IRS in the areas in this study, it was detected; a large number of infections and IRS was not associated with protection from malaria in univariate or multivariate models. This could be due to insecticide resistance as was documented by Kabula *et al.*, whose national surveillance demonstrated widespread resistance to pyrethroids among *Anopheles gambiae* across Tanzania [25]. It has also been reported that environmental management strategies to control breeding sites by either larval control or by other traditional methods have resulted in reduction of mosquito densities and malaria transmission [26, 27]. In Africa, larval source control is recommended where breeding sites are fixed, findable and few. This paper has shown that malaria risk is associated with proximity to known breeding sites, thus larval source control methods could be employed as an additional malaria control tool.

The finding that poor-quality housing is an independent risk factor for malaria infection in the second year agrees with previous studies [3, 28–32]. The presence of open eaves in house design and unscreened windows have been associated with increased risk for malaria infection, as the eaves are entry points to the household for malaria vectors [2, 4, 32, 33]. Quality of housing has been reported to influence the ease with which mosquitoes can enter and hide in a household and therefore contribute to malaria risk [33]. Although interventions to address quality of household construction as a malaria risk factor are complex and difficult to achieve, it might be important to add this component as an intervention for malaria control in public health programmes. In recent years, Rwanda has started a campaign to improve household structures by replacing thatched roofs with iron sheets, as malaria control strategy [34]. Improving everyone’s housing may be impossible but it might be cost effective to improve people’s housing within a hotspot.

## Conclusion

This paper demonstrates that living in a geographical cluster of households at high risk of malaria is an important independent risk factor for future malaria infection. In this analysis, living within a malaria hotspot as defined by SaTScan, showed a strong association with malaria infection in the subsequent year (OR 3.11, 95% CI 1.57, 6.18) independent of housing quality, proximity to breeding site, maternal education, distance from the health facility, and the use of both IRS and LLIN for vector control. This suggests that targeting hotspots with better coverage and improved malaria control strategies will likely result in a more cost-efficient use of resources to achieve malaria control and elimination. A remaining challenge is how malaria control programmes can detect these hotspots without having to conduct PCR prevalence or serological surveys.

Other risk factors, such as residing in households built from poor-quality materials, households situated near breeding sites and households that are far from health facilities, should also be explored to see if they can be used to lead a malaria surveillance officer to a hotspot.
